# Precision Pediatric Caries Diagnostics: Saliva-Check Mutans versus Culture for High-Density Streptococcus mutans Detection

**DOI:** 10.15190/d.2025.19

**Published:** 2025-12-31

**Authors:** Zeus Gómez Rodríguez, Saira Karina Ramírez Thome, Risk Díaz Castillejos, Eunice Daysi García-Reyes, Adrián Martínez-Vargas, Nahui Samanta Nájera-Segura, Efrén Emmanuel Jarquín González, Gilka Fernanda Nivon-Torres, Enrique Alfonso Acevedo Mascarua, Homero Caballero-Sánchez, Roberta Lizette Palacios-Cruz, Carlos Josué Solórzano-Mata, Taurino Amilcar Sosa-Velasco, César Zárate-Ortiz

**Affiliations:** ^1^Laboratorio de Bioquímica de Proteínas y Glicopatologías, Faculty of Dentistry, Universidad Autónoma "Benito Juárez" de Oaxaca, Oaxaca, Mexico; ^2^División de Estudios de Posgrado e Investigación, Tecnológico Nacional de México, Instituto Tecnológico del Valle de Etla, Abasolo S/N, Barrio del Agua Buena, Santiago Suchilquitongo, CP. 68030, Oaxaca, México; ^3^UNAM-UABJO Research Centre, Faculty of Medicine and Surgery, Universidad Autónoma Benito Juárez de Oaxaca (UABJO), Oaxaca 68120, Mexico; ^4^Dirección General de los Servicios de Salud de Oaxaca, Secretaria de Salud, Servicios de Salud de Oaxaca, Oaxaca, Mexico; ^5^Facultad de Ciencias Químicas, UABJO, 68120, Oaxaca, México, UABJO, Oaxaca 68120, Mexico; ^6^Facultad de Enfermería y Obstetricia Oaxaca (FAEO), UABJO, 68120, Oaxaca, México

**Keywords:** Dental caries, Streptococcus mutans, oral microbiome, point-of-care diagnostics, immuno-chromatographic assay, microbial dysbiosis, diagnostic thresholds, pediatric oral health, saliva-based diagnostics, precision dentistry.

## Abstract

The paradigm of dental medicine is shifting from a reactive surgical model to precision pediatric caries diagnostics, emphasizing early detection of pathogenic oral microbiota. Rapid point-of-care assays capable of identifying high-density Streptococcus mutans are critical to enable targeted intervention. This pilot study evaluated the diagnostic validity of a high- threshold monoclonal antibody-based lateral flow assay (Saliva-Check Mutans, SCM) relative to selective culture for identifying clinically meaningful S. mutans loads in children. Stimulated saliva samples were collected from 50 schoolchildren aged 9-13 years in Oaxaca, Mexico. Samples were analyzed using SCM and selective culture on Mitis Salivarius Agar (MSA), with presumptive S. mutans colonies confirmed biochemically. Selective culture identified 46% of participants as positive, whereas SCM detected 18% as positive. Relative to culture, SCM demonstrated 39.1% sensitivity (95% CI: 21.5%–60.1%), 100% specificity (95% CI: 87.5%–100%), and 100% positive predictive value (95% CI: 66.4%–100%), with no false positives observed. The results highlight the assay’s rule-in capability for high-density pathogenic loads (>10^5 CFU/mL). The diagnostic discordance reflects divergent analytical thresholds, termed the “Threshold Gap”. While SCM exhibits limited sensitivity for low-level colonization, its absolute specificity supports its use as a precision high-threshold triage tool, identifying pediatric patients with clinically significant S. mutans burdens who may benefit from intensified preventive strategies. Integration with culture or molecular approaches can enhance risk stratification and precision dentistry workflows.

## 1. INTRODUCTION

The traditional *"surgical model"* of dentistry, which focused predominantly on the restorative correction of cavitated lesions, has undergone a fundamental paradigm shift toward a *"medical model"*^1^. This modern approach prioritizes longitudinal disease management and the maintenance of ecological homeostasis within the oral cavity^2,3^. Central to this transition is the demand for rapid, point-of-care (POC) diagnostic tools that can accurately quantify microbial threats before irreversible structural damage occurs^4^. Recent advances in salivaomics and non-invasive monitoring have positioned saliva as a "diagnostic powerhouse", with more than 300 studies in 2024 alone highlighting its potential for precision health monitoring^5,6^.

Dental caries is currently understood through the Ecological Plaque Hypothesis, which posits that the disease is not caused by an exogenous pathogen but rather by a dysbiotic shift in the resident oral biofilm^7-9^. Under conditions of frequent dietary carbohydrate intake, the biofilm environment becomes persistently acidified, selecting for a unique consortium of acidogenic and aciduric pathobionts^10,11^. Recent systematic reviews (2025) confirm that while the oral microbiome is highly complex, *Streptococcus mutans* remains a primary microbial marker for caries risk due to its unparalleled ability to drive this ecological catastrophe^12-16^.

*Streptococcus mutans* serves as the prototype of this cariogenic profile, orchestrating a multifaceted attack on tooth mineral through several key virulence factors^13,17^. The acidogenicity of *S. mutans* is defined as the ability to rapidly ferment sucrose, glucose, and fructose into lactic acid via the glycolytic pathway^18,19^. This process can drop the local pH to approximately 4.2 within a 24-hour period^20^. To survive this self-induced toxicity, the organism exhibits extreme aciduricity^7,21^. It utilizes a membrane-bound F-ATPase system to pump excess hydrogen ions out of the cell. This mechanism allows the bacteria to maintain internal pH homeostasis in highly acidic environments^22^. Furthermore, recent transcriptomic analyses have elucidated the role of glucosyltransferases (*gtfB, gtfC, gtfD*) in extracellular polysaccharide (EPS) synthesis^23^. This EPS matrix acts as a molecular scaffold, promoting microbial aggregation and creating "acidic niches" that shield pathobionts from the buffering capacity of saliva.

Because *S. mutans* is a commensal member of the oral flora at low densities, its mere presence is not pathognomonic; rather, its pathogenicity is strictly density-dependent^24^. Accurate risk assessment requires distinguishing between various colonization states: a) Low-risk colonization (CFU/mL): Generally associated with a healthy, balanced microbiome where commensal species like *S. sanguinis* predominate^25^. b) Transitional/ Moderate risk (CFU/mL): A "grey zone" where microbial shifts are beginning, potentially leading to white spot lesions if protective factors are not reinforced^26^. c) High-risk pathogenic loads (CFU/mL): A consensus threshold where acid production often overwhelms natural re-mineralization, leading to cavitated lesions^27^.

Technological advances through 2024, notably machine learning–integrated caries risk algorithms and next-generation immunochromatography readers, have measurably increased the precision and clinical utility of point-of-care caries forecasting^28^. Addressing the systemic 'valley of death' in Mexican biomedical research requires overcoming translational barriers to resolve the Threshold Gap between analytical detection and clinical decision limits^29^. In this context, we evaluated the SCM monoclonal antibody lateral flow assay against selective culture, the accepted laboratory "gold standard"^30^, with three primary aims: (1) quantify diagnostic accuracy, including sensitivity, specificity, and predictive values across clinically relevant thresholds; (2) assess reproducibility and preanalytical influences that may bias chairside results; and (3) determine how high-threshold diagnostics perform when integrated into contemporary, algorithm-driven risk models.

**Figure 1:**
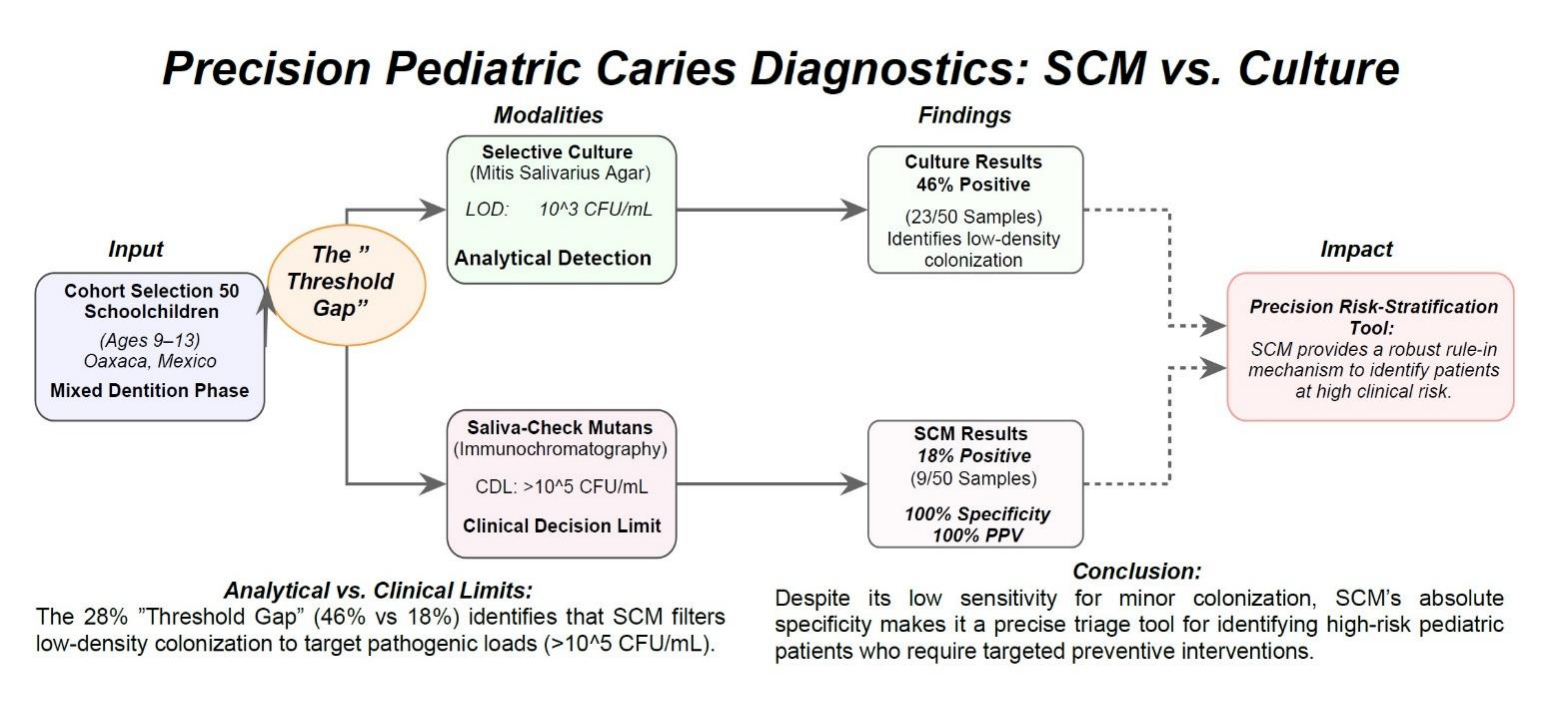
Visual Abstract: Precision Pediatric Caries Diagnostics

Prior work has reported a characteristic performance profile for SCM type assays, namely high sensitivity with only moderate specificity, which underscores the potential for false positive classification if used in isolation^31^. Accordingly, this study situates SCM performance within the contemporary literature and explores whether coupling monoclonal antibody assays with automated readers and machine learning risk stratification can shift their role from a binary screening device to a calibrated component of precision dentistry workflows (see Figure 1 – visual abstract).

## 2. MATERIAL AND METHODS

The study design employed a cross-sectional diagnostic validation following the 2024-2025 STARD (Standards for Reporting Diagnostic Accuracy) guidelines for clinical microbiology.

### 2.1 Population and Demographic Selection

The cohort comprised 50 schoolchildren aged 9–13 years in Oaxaca, Mexico (mean age 10.46 ± 1.15 years; n = 50). Participants were in the mixed dentition phase, a critical window for oral microbiome stabilization as permanent teeth erupt into a pre‑colonized environment^32,33^. Subjects who had received antibiotic therapy within the preceding 30 days were excluded to avoid suppression of the oral flora^34^. The sample had a balanced sex distribution (24 males, 48%; 26 females, 52%), providing an appropriate population for assessment of cariogenic activity.

### 2.2 Salivary Sampling and Point-of-Care Assay

Stimulated whole saliva was collected after ≥60 minutes abstinence from food, drink, and oral hygiene by instructing participants to chew paraffin wax for 60–90 seconds and expectorate into a sterile container; samples were processed immediately. Specimens were handled according to the Saliva Check Mutans (SCM) (GC America) manufacturer protocol^30,31,35^: sequential addition of NaOH for alkaline lysis, citric/organic acid for neutralization to the kit colorimetric end point, and a final neutralizing buffer to condition the sample for lateral flow migration. Processed aliquots were applied to the SCM lateral flow cassette and read at the manufacturer specified time (≈15 minutes); indeterminate results were repeated once. Operators were trained and blinded to culture results; lot numbers and visual end points were recorded. SCM outputs (rapid, semi quantitative) were compared with selective culture on mitis salivarius bacitracin agar as the reference standard. Limitations included preanalytical variability (stimulation method, timing), potential incomplete neutralization affecting antibody binding, semi quantitative output, and possible cross reactivity, all of which were acknowledged and controlled where feasible.

### 2.3 Reference Standard: Selective Microbiological Culture

Samples were inoculated onto Mitis Salivarius Agar (MSA) with bacitracin for selective recovery of Streptococcus mutans. A calibrated 1 µL inoculation loop was used to plate saliva aliquots; plates were incubated at 37°C for 48–96 hours to allow colony maturation. Presumptive S. mutans colonies were identified by characteristic morphology and confirmed by mannitol and sorbitol fermentation. Using a 1 µL plating volume, the analytical lower limit of detection (LOD) corresponds to approximately 1,000 CFU/mL (plated colony count × 1,000). Culture based quantification served as the reference standard for comparing the SCM clinical decision limit (CDL) with the analytical LOD^36,37^. Diagnostic performance was evaluated using a contingency table to calculate sensitivity, specificity, positive predictive value (PPV), and negative predictive value (NPV). Data were analyzed to identify the "Threshold Gap" between the culture LOD ( CFU/mL) and the SCM CDL ( CFU/mL)^38^.

## 3. RESULTS

### 3.1 Diagnostic Performance

A total of 50 stimulated saliva samples were collected from schoolchildren aged 9–13 years and analyzed using both an immunochromatographic assay (Saliva-Check Mutans, SCM) and selective culture as the reference method. All samples yielded sufficient material for parallel testing and were included in the final analysis. Using SCM, 9 of 50 samples (18%) were classified as positive for *Streptococcus mutans*. In contrast, selective culture identified 23 of 50 samples (46%) as positive. Approximate 95% confidence intervals for sensitivity, specificity, positive predictive value, and negative predictive value were calculated using binomial methods and are reported to provide an estimate of precision given the modest sample size.

*Selective culture results and analytical characteristics. *Saliva samples were plated on Mitis Salivarius Agar supplemented with bacitracin and incubated at 37°C for 48–96 hours. Inoculation was performed using a 1 µL calibrated loop, resulting in an estimated analytical LOD of approximately 1,000 CFU/mL, calculated as the observed colony count multiplied by 1,000. MSA supplemented with bacitracin was selected as the reference method due to its widespread use in clinical and public health microbiology laboratories, compatibility with prior SCM validation studies, and feasibility in resource-limited settings. Although alternative media and molecular assays may provide higher analytical recovery, the use of a low-threshold culture reference intentionally accentuates the analytical–clinical ‘Threshold Gap’ that is central to the present study’s objective.

Presumptive *S. mutans* colonies were identified based on characteristic morphology and subsequently confirmed by mannitol and sorbitol fermentation tests^39^. Using these criteria, 23 samples (46%) demonstrated confirmed growth of *S. mutans* above the analytical LOD.

*Concordance between SCM and selective culture. *Cross-classification of SCM results against selective culture demonstrated that 9 samples were true positives (TP)**, **14 were false negatives (FN), 27 were true negatives (TN), and no false positives (FP) were observed**. **These concordance data are summarized in Table 1.

**Table 1:** Contingency table for Saliva-Check Mutans versus selective culture (n=50)

	Culture positive	Culture negative	Total
**SCM positive**	9 (TP)	0 (FP)	9
**SCM negative**	14 (FN)	27 (TN)	41
**Total**	23	27	50

*Diagnostic performance of Saliva-Check Mutans. *Using selective culture as the reference standard, SCM demonstrated a sensitivity of 39.13% (9/23) and a specificity of 100% (27/27)**.** SCM demonstrated a sensitivity of 39.13% (95% CI: 21.51–59.41%) and a specificity of 100% (95% CI: 87.23–100.00%). The positive predictive value was 100.00% (95% CI: 66.37–100.00%) and the negative predictive value was 65.85% (95% CI: 49.41–79.92%).The overall diagnostic accuracy of SCM was approximately 72%. Approximate 95% confidence intervals for sensitivity, specificity, positive predictive value, and negative predictive value were calculated using standard binomial methods and are reported to provide an estimate of precision given the modest sample size. Notably, no false-positive SCM results were detected, whereas 14 samples identified as positive by selective culture were classified as negative by SCM, accounting for the reduced sensitivity observed (Table 2).

**Table 2:** Table 2. Diagnostic performance of Saliva-Check Mutans (SCM) using selective culture as reference standard

Metric	Value	95% Confidence Interval (CI)
**Sensitivity**	39.13% (9/23)	21.51%–59.41%
**Specificity**	100% (27/27)	87.23%–100.00%
**Positive predictive value**	100% (9/9)	66.37% – 100.00%
**Negative predictive value**	65.85% (27/41)	49.41% – 79.92%
**Overall accuracy**	72% (36/50)	57.51% – 83.77%

*Analytical Thresholds and Comparative Performance. *The analytical LOD of selective culture was approximately 1,000 CFU/mL, determined by the use of a calibrated 1 µL inoculation loop and the requirement for visible colony formation. This reflects the high sensitivity of culture for detecting low-density Streptococcus mutans colonization. In contrast, the CDL of the SCM assay is not explicitly defined but is inferred to be substantially higher based on observed discordance, defining the “Threshold Gap” between the two methods.

To contextualize these findings, we compared results with an independent dataset (n =25) evaluating SCM, Caries Risk Test (CRT), and CariScreen ATP bioluminescence using conventional culture^31^. In the contextual cohort, culture identified 52% of subjects as high risk, and no false-negative classifications were observed. As summarized in Table 3, SCM showed higher sensitivity (88.42%) but lower specificity (75.00%) compared with our study (39.13% sensitivity; 100% specificity). McNemar’s chi-square test revealed no statistically significant discordance between chairside assays and culture in the contextual dataset (P ≥ 0.47), in contrast to the pronounced Threshold Gap observed in the Oaxaca cohort. These results indicate that SCM performance is primarily influenced by population risk distribution and the characteristics of the reference standard, rather than by intrinsic assay variability.

**Table 3:** Table 3. Unified Comparative Performance of Chairside Assays Across Independent Cohorts* Values are reported as percentages. Conventional culture served as the reference standard. PPV, positive predictive value; NPV, negative predictive value. The current study used Mitis Salivarius Agar (MSA) with biochemical confirmation via mannitol and sorbitol fermentation as the selective culture reference standard. Babu et al., 2019 employed Mitis Salivarius Bacitracin (MSB) agar as the conventional selective culture reference, with bacitracin providing selective inhibition of non-mutans streptococci^31^. *Note: The diagnostic performance of SCM is population-dependent; the observed sensitivity and specificity are functions of the baseline caries risk and microbial density distribution within the study cohort, rather than intrinsic assay instability. All 95% Confidence Intervals (CI) were calculated using the Clopper-Pearson exact method.

Study / Assay	Cohort	Sensitivity (95% CI)	Specificity (95% CI)	PPV (95% CI)	NPV (95% CI)	Accuracy (95% CI)
**SCM (Current Study)**	Oaxaca (n=50)	39.13% (21.51-59.41)	100.00% (87.23-100.00)	100.00% (66.37-100.00)	65.85% (49.41-79.92)	72.00% (57.51-83.77)
**SCM 31**	Babu et al., 2019 (n=25)	88.42% (61.65-98.43)	75.00% (42.81-94.51)	81.25% (54.35-95.95)	100.00% (63.06-100.00)	88.00% (68.78-97.45)
**CRT 31**	Babu et al., 2019 (n=25)	93.14% (66.13-99.82)	91.67% (61.52-99.79)	92.86% (66.13-99.82)	100.00% (73.54-100.00)	96.00% (79.62-99.90)
**CariScreen ATP31**	Babu et al., 2019 (n=25)	92.86% (66.13-99.82)	91.67% (61.52-99.79)	92.86% (66.13-99.82)	91.67% (61.52-99.79)	96.00% (79.62-99.90)

Time-to-result varied markedly: CariScreen ATP produced results in under one minute, SCM in ~15 minutes, and culture required 24-96 hours. Only culture enabled quantitative bacterial assessment, whereas chairside assays provided qualitative or threshold-based outputs. While ATP bioluminescence assays provide rapid (<1 minute) assessment of total biofilm metabolic activity, they lack taxonomic specificity^31^. In contrast, the SCM immunochromatographic assay provides species-specific targeting of S*. *mutans, the primary driver of the acidogenic shift in the oral microbiome^40^. Recent literature (2024–2025) emphasizes that while total biomass measured by ATP is a general marker of oral hygiene, identifying the presence of high-density pathobionts such as S. mutans is critical for precision dentistry and for implementing targeted antimicrobial or preventive therapies^7,13,15^. Scientifically, SCM is preferable in clinical scenarios where the identification of the biological driver of dysbiosis is more relevant than the overall metabolic state^41^. These findings support SCM as a rapid, high-threshold “rule-in” tool for point-of-care risk stratification when integrated into clinical workflows. The observed performance differences and threshold-dependent discordance motivate a deeper exploration of the biological, methodological, and clinical factors influencing SCM utility, as discussed in the following section.

## 4. DISCUSSION

The marked discrepancy between the diagnostic performance of SCM observed in our Oaxaca, Mexico pediatric cohort and results reported in prior studies motivates the concept introduced here as the “Threshold Gap”^31^. In our cohort, SCM demonstrated a sensitivity of 39.13% and a specificity of 100% relative to selective culture, whereas Babu et al. reported substantially higher sensitivity (88.42%) accompanied by lower specificity (75.00%)^31^. These divergent outcomes indicate that SCM performance is highly contingent upon both the baseline caries risk of the tested population and the analytical characteristics of the reference (“gold standard”) method used for comparison.

The low sensitivity observed in the Oaxaca cohort indicates that SCM frequently failed to detect low-density *Streptococcus mutans* colonization that was otherwise identified by selective culture. This finding is consistent with observations by Saravia et al., who reported perfect agreement (Kappa=1) between SCM and culture when evaluating individuals preclassified as high-risk^42^. Collectively, these data suggest that SCM is not intended as an analytical presence/absence assay but rather as a detector of high-density pathogenic loads exceeding a defined clinical decision limit. The elevated sensitivity reported by Babu et al. likely reflects cohort enrichment for individuals with microbial burdens above this threshold^31^. Conversely, in low-risk populations, SCM’s analytical cutoff yields an apparent loss of sensitivity despite appropriate performance for its intended clinical application.

The 100% specificity observed in our cohort contrasts sharply with values reported by Voelker et al. (25%) and Babu et al. (75%)^31,43^. From a clinical and public health perspective, high specificity represents a critical advantage, as it minimizes false-positive classifications and reduces the risk of unnecessary or overly aggressive interventions. Given the multifactorial nature of dental caries, in which salivary buffering capacity, salivary flow rate, dietary patterns, and host-related factors collectively modulate disease risk^44^, a highly specific SCM functions effectively as a rule-in test by selectively identifying individuals with genuinely elevated pathogenic burden, thereby guiding intensive preventive strategies while minimizing unnecessary interventions and microbiome disruption in moderate-risk carriers.

Reported diagnostic accuracy is strongly influenced by the choice of reference medium. In the present study, Mitis Salivarius Bacitracin (MSB) agar was used for selective culture. Prior evaluations indicate that MSB exhibits lower recovery rates for *S. mutans* compared with alternative media such as TYCSB or HLR-S^45^. Reduced recovery by the reference standard may bias performance estimates by underestimating sensitivity and inflating specificity of the index test. Methodological heterogeneity in reference standards therefore contributes substantially to interstudy variability. In this context, Damle et al. have argued that molecular approaches, including PCR-based assays, provide superior analytical precision and speed, and may represent a more appropriate benchmark for future validation studies of chairside diagnostics^46^. Adoption of molecular reference standards could help resolve the “Threshold Gap” inherent in antigen-based detection systems.

The clinical relevance of detecting high *S. mutans* loads extends beyond species presence. High-density *S. mutans* facilitates biofilm maturation and the incorporation of additional pathogenic taxa through glucosyltransferase-mediated mechanisms (GtfB and GtfC), thereby amplifying cariogenic potential^47^. In this context, SCM may function as a sentinel marker of broader dysbiotic shifts within the oral microbiome. Nevertheless, because SCM measures antigenic presence of a single species, its interpretive value is maximized when integrated into multifactorial risk models. Morou-Bermudez et al. emphasize that metabolic parameters, including the balance between acidogenic and alkali-generating pathways (sugar versus urea metabolism), are equally critical determinants of caries risk^48^. Accordingly, SCM should be viewed as one component within a composite biological framework rather than a standalone diagnostic.

Several limitations warrant consideration. First, although selective culture was performed using Mitis Salivarius Agar supplemented with bacitracin, differences in formulation, incubation conditions, and confirmatory workflows relative to standardized MSB-based protocols used in other studies^31^ may influence comparative diagnostic performance metrics. In the present study, biochemical confirmation using mannitol and sorbitol fermentation was required to ensure accurate identification of *S. mutans*, which may affect interstudy comparability of culture positivity rates. Second, while the sample size in the present study (n=50) is appropriate for a diagnostic validation focused on high-threshold detection, it limits the broader epidemiological generalizability of prevalence estimates. For comparison, Babu et al. evaluated SCM performance in a smaller cohort (n=25), yet reported higher sensitivity and lower specificity, highlighting the influence of both cohort size and population risk distribution on measured diagnostic metrics^31^. Nevertheless, given the high background prevalence of *S. mutans* in this pediatric age group and the pronounced Threshold Gap observed between SCM and selective culture in Oaxaca, our sample provided sufficient statistical resolution to characterize the assay’s specificity and rule-in performance. Future multicenter and longitudinal studies with larger and more diverse populations will be essential to validate these findings across varying epidemiological contexts and to refine SCM clinical decision thresholds. Third, the SCM assay does not provide quantitative bacterial counts beyond its decision threshold, nor does it offer insight into microbial viability or broader community structure. Finally, interstudy comparisons are constrained by heterogeneity in sampling protocols, cohort stratification, reference standards, and analytical thresholds, complicating direct numerical comparisons across reports.

### 4.2. Clinical and research implications

From a clinical standpoint, SCM should be positioned as a high-threshold, point-of-care “rule-in” assay to identify pediatric patients with clinically meaningful Streptococcus mutans burdens who may benefit from intensified preventive strategies. Its primary utility lies in guiding targeted interventions while minimizing overtreatment in individuals with low or moderate colonization. For population-level surveillance or detection of low-density colonization, quantitative culture or molecular diagnostics remain indispensable. Future research should emphasize standardized reference methods, explicit risk stratification, and integration of SCM with metabolic and host-based indicators to enhance precision in caries risk assessment. By situating SCM within a multifactorial diagnostic framework, clinicians can leverage its specificity without compromising broader preventive strategies, advancing the goals of precision dentistry.

## 5. CONCLUSION

This analysis demonstrates that SCM functions as a specialized high-threshold discriminatorrather than a universal screening tool. The apparent discordance between the low sensitivity observed in the Oaxaca, Mexico cohort and higher sensitivities reported elsewhere is attributable to differences in population risk profiles, analytical thresholds, and reference standards. While prior studies highlight the potential for false positives or reduced specificity in certain settings^31,43^, our findings underscore SCM’s value as a highly specific “rule-in” diagnostic in pediatric populations. As a pilot validation study, our results highlight that its limited sensitivity to low-level colonization should be interpreted not as technical failure but as an intentional clinical filter designed to target high-risk pathogenic loads, underscoring the need for larger, prospective studies. SCM offers unique species-specific targeting that complements rapid metabolic assays, such as ATP bioluminescence, which do not identify specific pathobionts. However, the continued evolution of precision dentistry will require replacing culture-based benchmarks with standardized molecular approaches, such as qPCR, to resolve the "Threshold Gap". When integrated with quantitative, metabolic, and molecular approaches, SCM may contribute meaningfully to precision dentistry frameworks aimed at minimizing overtreatment while targeting those at greatest risk.
